# Supplementary material for: Identification and Characterization of Antibiotic-Resistant, Gram-Negative Bacteria Isolated from Korean Fresh Produce and Agricultural Environment
Source: Microorganisms. 2023 May 8;11(5):1241. doi: 10.3390/microorganisms11051241 (PMC10222586; doi:10.3390/microorganisms11051241)
Supplement: Supplementary file 1 [file microorganisms-11-01241-s001.zip › microorganisms-2326321-supplementary.pdf]

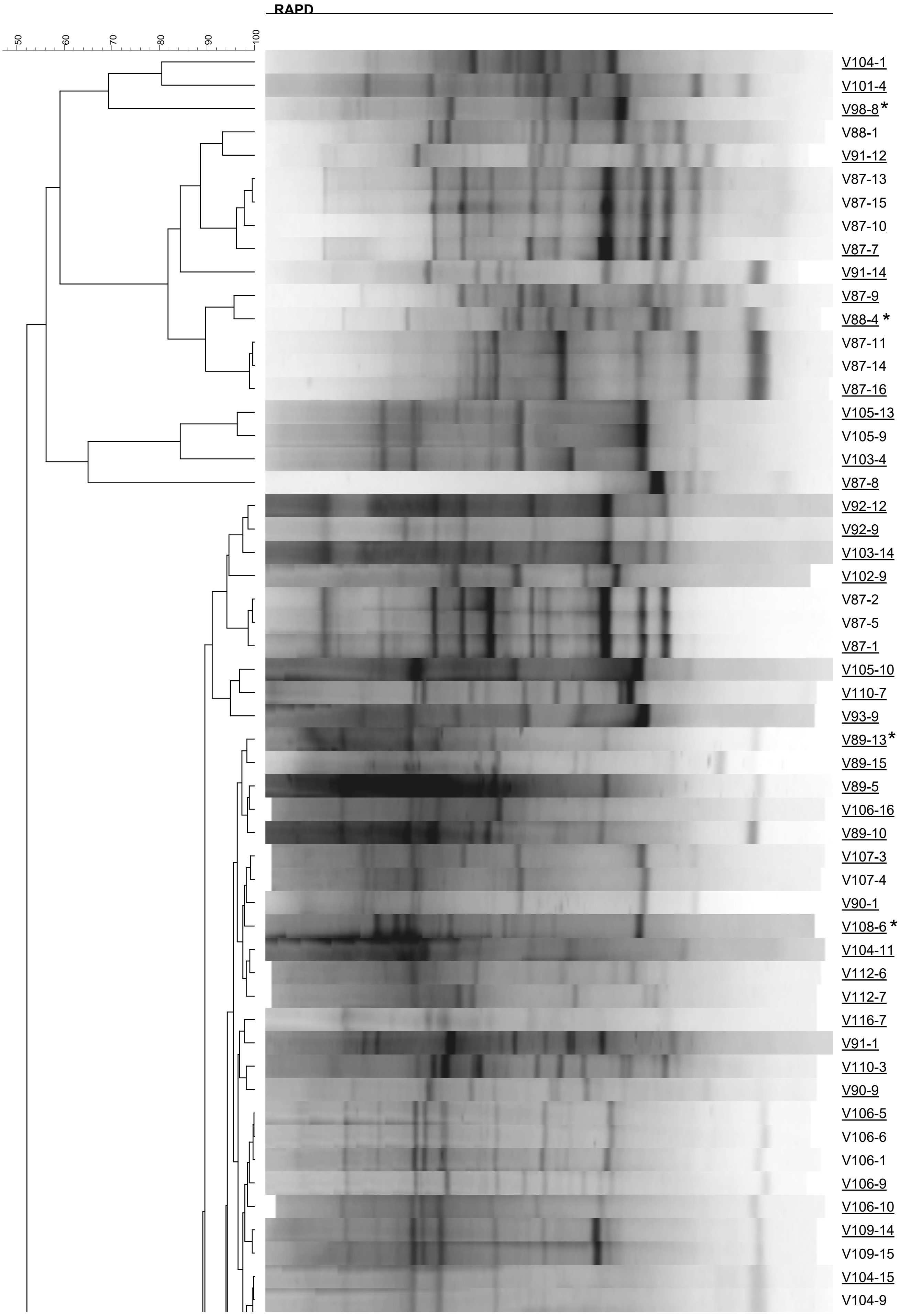
RAPD
50
60
70
80
90
100
V104-1
V101-4
V98-8 *
V88-1
V91-12
V87-13
V87-15
V87-10
V87-7
V91-14
V87-9
V88-4 *
V87-11
V87-14
V87-16
V105-13
V105-9
V103-4
V87-8
V92-12
V92-9
V103-14
V102-9
V87-2
V87-5
V87-1
V105-10
V110-7
V93-9
V89-13 *
V89-15
V89-5
V106-16
V89-10
V107-3
V107-4
V90-1
V108-6 *
V104-11
V112-6
V112-7
V116-7
V91-1
V110-3
V90-9
V106-5
V106-6
V106-1
V106-9
V106-10
V109-14
V109-15
V104-15
V104-9

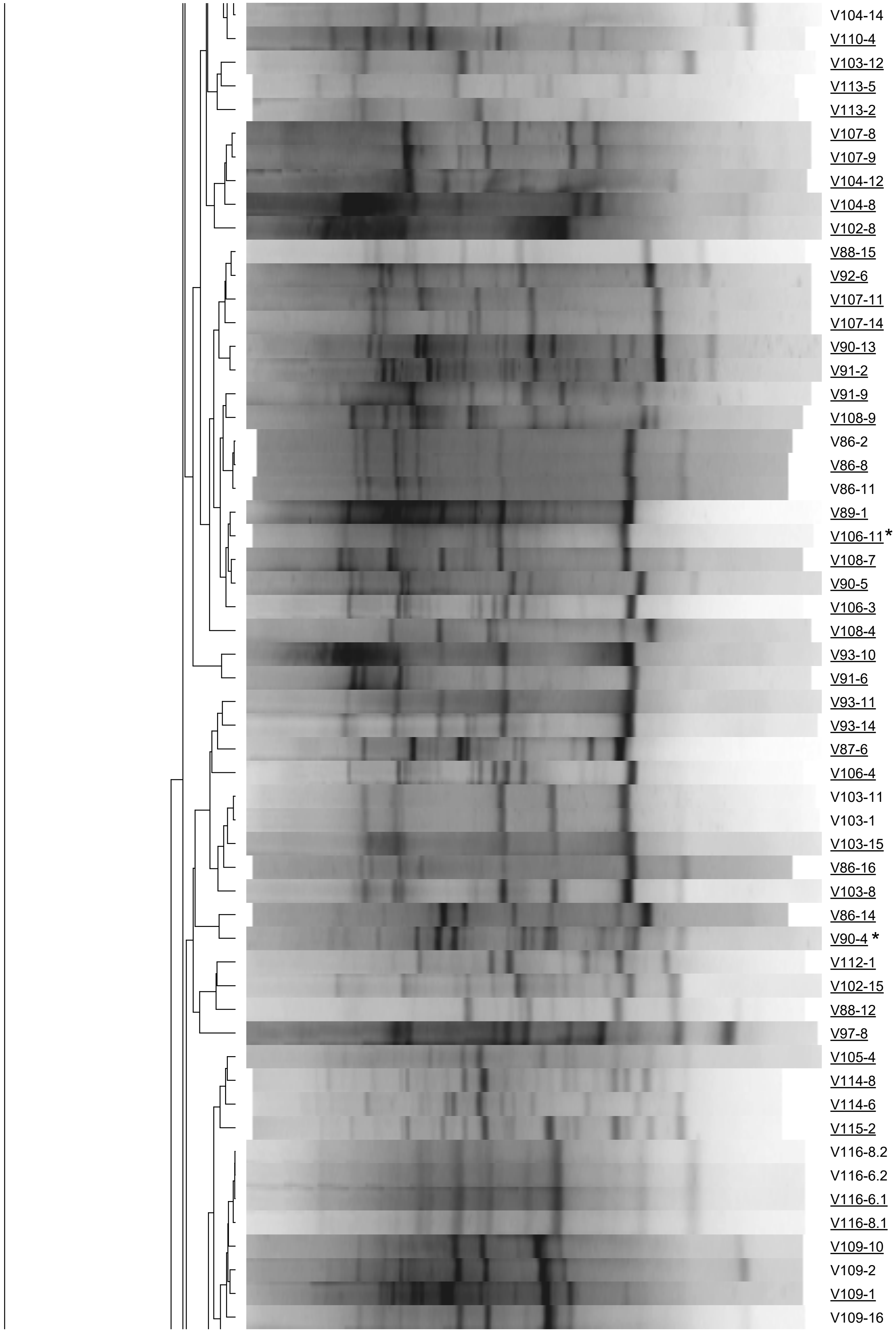

V104-14
V110-4
V103-12
V113-5
V113-2
V107-8
V107-9
V104-12
V104-8
V102-8
V88-15
V92-6
V107-11
V107-14
V90-13
V91-2
V91-9
V108-9
V86-2
V86-8
V86-11
V89-1
V106-11*
V108-7
V90-5
V106-3
V108-4
V93-10
V91-6
V93-11
V93-14
V87-6
V106-4
V103-11
V103-1
V103-15
V86-16
V103-8
V86-14
V90-4 *
V112-1
V102-15
V88-12
V97-8
V105-4
V114-8
V114-6
V115-2
V116-8.2
V116-6.2
V116-6.1
V116-8.1
V109-10
V109-2
V109-1
V109-16

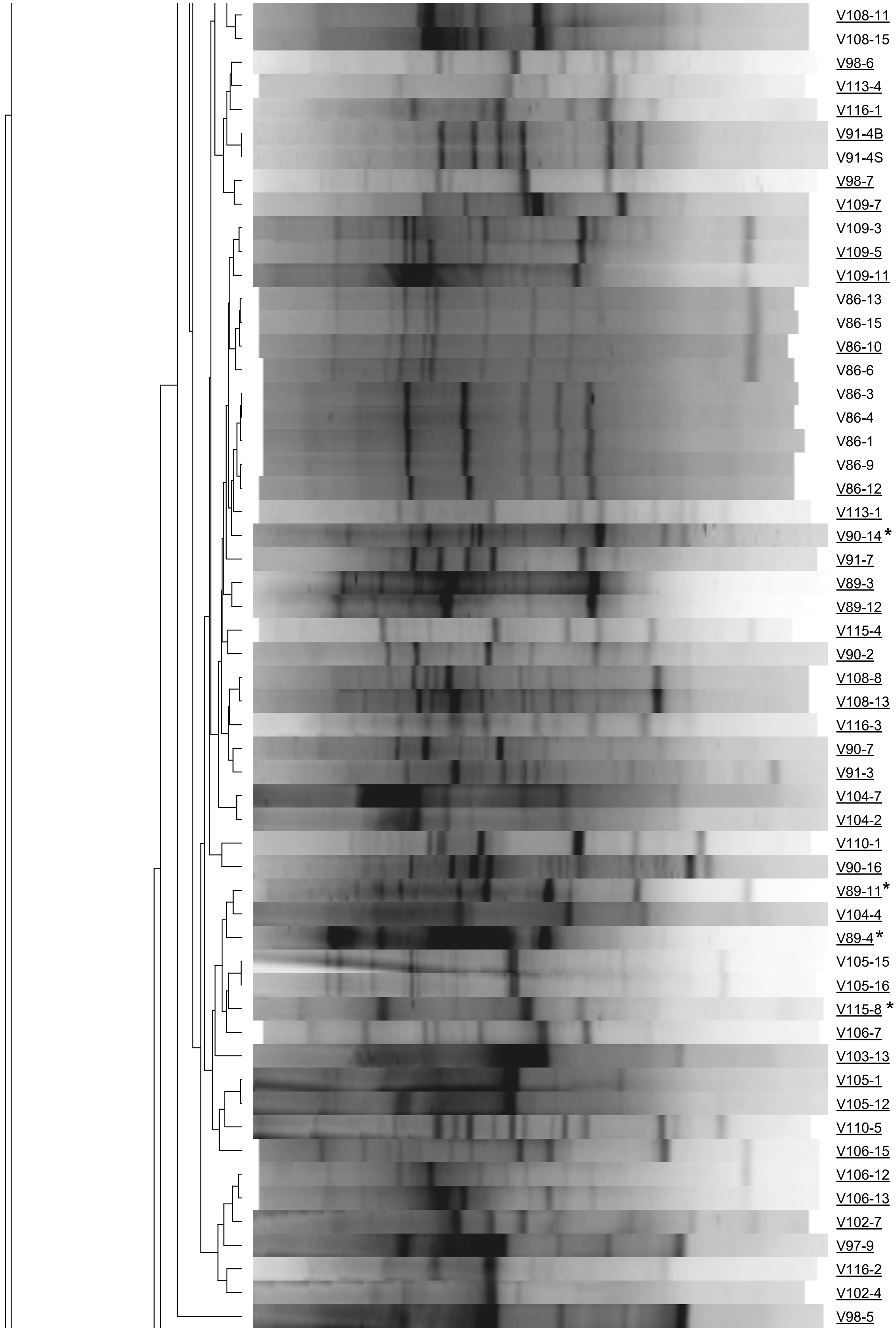
V108-11
V108-15
V98-6
V113-4
V116-1
V91-4B
V91-4S
V98-7
V109-7
V109-3
V109-5
V109-11
V86-13
V86-15
V86-10
V86-6
V86-3
V86-4
V86-1
V86-9
V86-12
V113-1
V90-14 *
V91-7
V89-3
V89-12
V115-4
V90-2
V108-8
V108-13
V116-3
V90-7
V91-3
V104-7
V104-2
V110-1
V90-16
V89-11*
V104-4
V89-4*
V105-15
V105-16
V115-8 *
V106-7
V103-13
V105-1
V105-12
V110-5
V106-15
V106-12
V106-13
V102-7
V97-9
V116-2
V102-4
V98-5

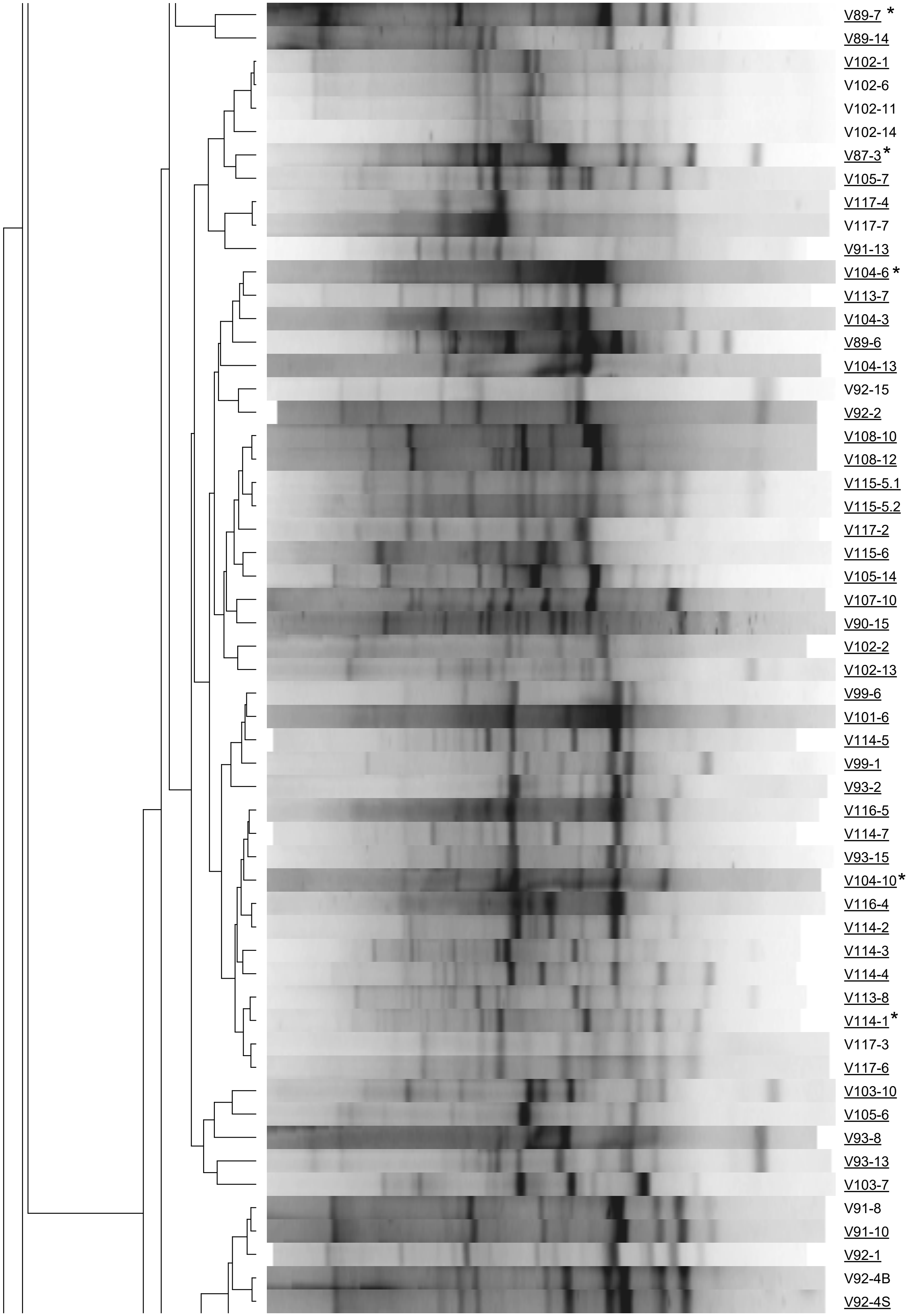
V89-7 *
V89-14
V102-1
V102-6
V102-11
V102-14
V87-3*
V105-7
V117-4
V117-7
V91-13
V104-6*
V113-7
V104-3
V89-6
V104-13
V92-15
V92-2
V108-10
V108-12
V115-5.1
V115-5.2
V117-2
V115-6
V105-14
V107-10
V90-15
V102-2
V102-13
V99-6
V101-6
V114-5
V99-1
V93-2
V116-5
V114-7
V93-15
V104-10*
V116-4
V114-2
V114-3
V114-4
V113-8
V114-1*
V117-3
V117-6
V103-10
V105-6
V93-8
V93-13
V103-7
V91-8
V91-10
V92-1
V92-4B
V92-4S

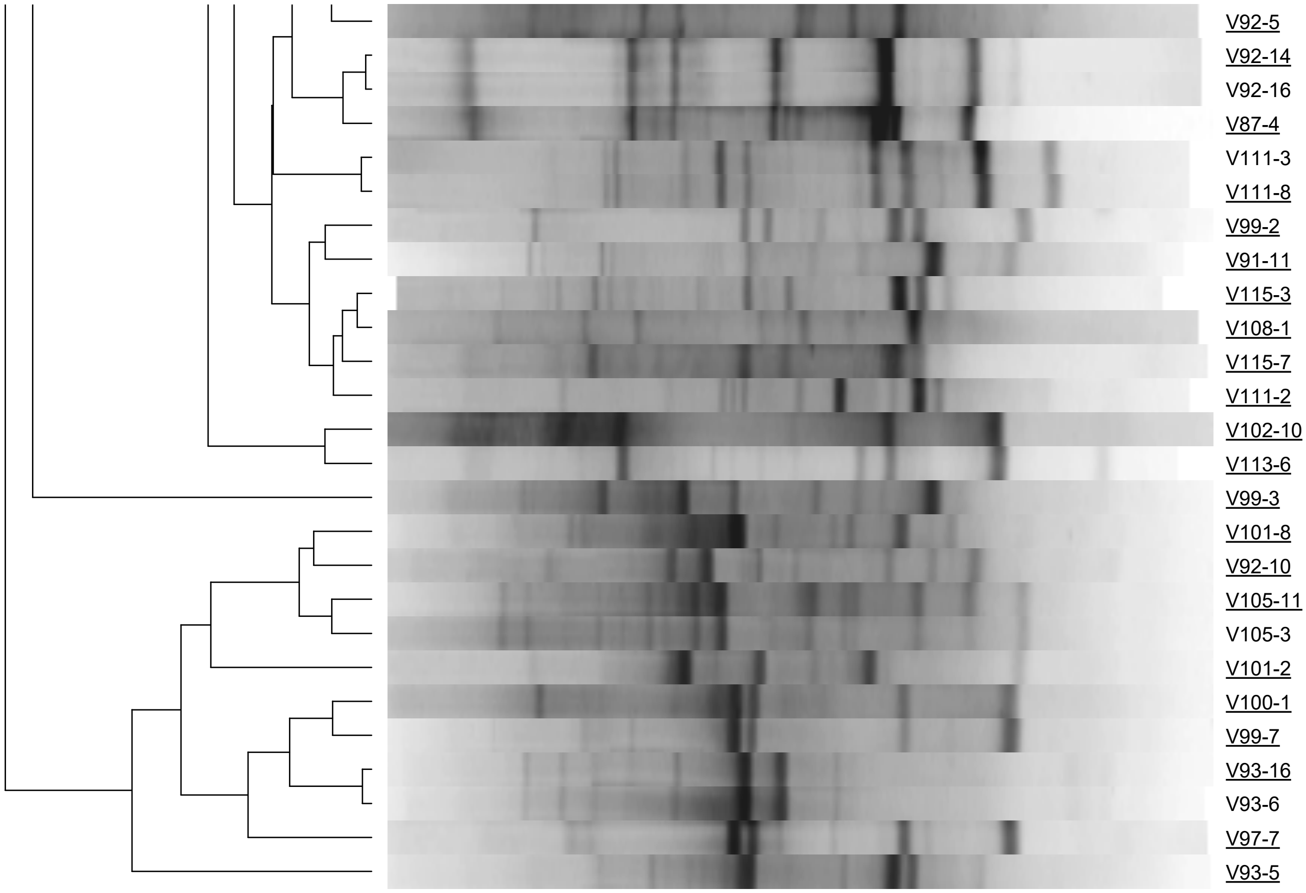

Figure S1: Dendrogram obtained by UPGMA clustering using the Pearson correlation coefficient of RAPD-PCR fingerprints of 248 Gram-negative isolates from lettuces and different fertilized soil samples. Selected strains for 16S rRNA gene sequencing were marked with an under bar. *; selected isolates for whole genome sequencing.
